# Induced Sputum MMP-1, -3 & -8 Concentrations during Treatment of Tuberculosis

**DOI:** 10.1371/journal.pone.0061333

**Published:** 2013-04-22

**Authors:** Cesar A. Ugarte-Gil, Paul Elkington, Robert H. Gilman, Jorge Coronel, Liku B. Tezera, Antonio Bernabe-Ortiz, Eduardo Gotuzzo, Jon S. Friedland, David A. J. Moore

**Affiliations:** 1 Instituto de Medicina Tropical Alexander Von Humboldt, Universidad Peruana Cayetano Heredia, Lima, Perú; 2 Department of Infectious Diseases and Immunity, Imperial College London, London, United Kingdom; 3 Imperial College Wellcome Trust Centre for Clinical Tropical Medicine, London, United Kingdom; 4 Clinical and Experimental Sciences, University of Southampton, Southampton, United Kingdom; 5 Laboratorios de Investigación y Desarrollo, Universidad Peruana Cayetano Heredia, Lima, Perú; 6 Department of International Health, Johns Hopkins Bloomberg School of Public Health, Baltimore, Maryland, United States of America; 7 CRONICAS Center of Excellence in Chronic Diseases, Universidad Peruana Cayetano Heredia, Lima, Perú; 8 Epidemiology Unit, School of Public Health and Administration, Universidad Peruana Canyetano Heredia, Lima, Perú; 9 LSHTM TB Centre and Department of Clinical Research, London School of Hygiene and Tropical Medicine, London, United Kingdom; Institut de Pharmacologie et de Biologie Structurale, France

## Abstract

**Introduction:**

Tuberculosis (TB) destroys lung tissues and this immunopathology is mediated in part by Matrix Metalloproteinases (MMPs). There are no data on the relationship between local tissue MMPs concentrations, anti-tuberculosis therapy and sputum conversion.

**Materials and Methods:**

Induced sputum was collected from 68 TB patients and 69 controls in a cross-sectional study. MMPs concentrations were measured by Luminex array, TIMP concentrations by ELISA and were correlated with a disease severity score (TBscore). 46 TB patients were then studied longitudinally at the 2nd, 8th week and end of treatment.

**Results:**

Sputum MMP-1,-2,-3,-8,-9 and TIMP-1 and -2 concentrations are increased in TB. Elevated MMP-1 and -3 concentrations are independently associated with higher TB severity scores (p<0.05). MMP-1, -3 and -8 concentrations decreased rapidly during treatment (p<0.05) whilst there was a transient increase in TIMP-1/2 concentrations at week 2. MMP-2, -8 and -9 and TIMP-2 concentrations were higher at TB diagnosis in patients who remain sputum culture positive at 2 weeks and MMP-3, -8 and TIMP-1 concentrations were higher in these patients at 2nd week of TB treatment.

**Conclusions:**

MMPs are elevated in TB patients and associate with disease severity. This matrix-degrading phenotype resolves rapidly with treatment. The MMP profile at presentation correlates with a delayed treatment response.

## Introduction

Tuberculosis (TB) remains a global pandemic, with around 8.7 million new cases in 2011 [1]. Multi and extensively drug resistant TB is increasing world-wide and although some new drugs are emerging [2], new approaches to therapy are required which depend on improved understanding of pathophysiology. In patients with active pulmonary disease, there is lung tissue damage, most characteristically with cavitation [3]. Such tissue damage facilitates spread of *M. tuberculosis* (Mtb). Lung tissue destruction is in part a consequence of Matrix Metalloproteinase (MMP) activity [4]. MMPs are zinc-dependent proteases which collectively can degrade all components of the extracellular matrix and are involved in remodelling and repair of diverse tissues [5], [6], [7]. Increased MMP activity is associated with inflammation in diverse infections [8], [9], [10] as well as in non-infective pulmonary diseases such as asthma and COPD [11].

We and others have demonstrated that MMPs are key in destruction of the lung interstitial matrix in TB and may have a role in regulating leukocyte migration to the granuloma [4], [12]. Mtb may drive MMP-1, -3, -7 and -9 secretion from monocytes, macrophages or pulmonary epithelial cells, suggesting that multiple proteases may contribute to pathology in TB [13], [14], [15], [16]. Relatively few small, focused clinical studies have demonstrated increased MMP concentrations in TB patients usually with minimal analysis of clinical data [4], [17]. Furthermore, no longitudinal investigation of changes in secretion of MMPs and their specific Tissue Inhibitors of Metalloproteinases (TIMPs) during TB treatment has been performed. In addition, the relationship between time to culture conversion on TB treatment and MMP activity is unknown.

In this study, we first compared induced sputum MMP and TIMP concentrations in TB patients with healthy controls in a prospectively recruited, carefully clinically characterised cohort of HIV negative patients. Next, we investigated alterations in sputum MMP and TIMP concentrations during the 6 months treatment period and their relation to sputum culture conversion.

## Methods

### Study design

First, a cross-sectional evaluation of TB patients and controls was performed. Next, from these TB patients, a longitudinal, prospective follow-up was performed during the standard 6-month period of treatment for TB.

### Population recruitment

The recruitment occurred between August 2009 to November 2010. . Study participants were recruited in health centres in Lima. Inclusion criteria for TB participants were: microbiologically confirmed diagnosis of TB (positive sputum smear microscopy and/or positive TB culture), age ≥18 years, cough with sputum production and no prior history of TB or TB treatment.

Controls were recruited among the relatives who were accompanying pregnant women or children for routine medical visits and/or accompanying patients with non-related TB disease. The inclusion criteria for controls were: over 18 years of age, had no symptoms associated with TB (cough for more than 14 days, night sweating and weight loss), no known TB contact, a normal chest radiograph and negative sputum TB culture.

Exclusion criteria for both groups were immunological compromise including HIV infection and current treatment with corticosteroids and/or any other immunomodulatory drugs, as well as any conditions where sputum induction is contra-indicated including asthma, chronic obstructive pulmonary disease or oxygen saturations <94%. Any form of drug resistant TB also excluded a patient from the study.

### Field Study procedures

Eligible patients gave informed consent and underwent a clinical and epidemiological evaluation. To evaluate the severity of the disease, we used the TBscore [18]. TBscore is a simple clinical evaluation score used for clinical monitoring of TB patients in low-resource settings, which may be used to predict mortality risk. Mortality and disease severity are increased in patients with a TBscore greater than 8. In addition, digital chests X-rays (CXR) were obtained and were scored for degree of pulmonary infiltration using ImageJ analysis [17]. Blood was taken for HIV ELISA testing. Follow-up evaluation of TB patients was performed at 2, 8 and 24 weeks (at the end of therapy) after starting TB treatment and at each time point, an induced sputum sample was taken and the TBscore re-evaluated.

Sputum induction was performed to obtain consistent samples from the bronchial tree. The procedure for sputum induction (SI) was in a designated sputum collection cabin, situated outside of the main clinical facility with an open roof and good ventilation. Staff infection control protection measures included use of appropriate respiratory protection masks (N95) and minimizing the time spent in the room during the procedure. For each SI procedure, 30 mL of 3% hypertonic saline was administered via a mouthpiece using a nebulizer NA180 (Aspen, Buenos Aires, Argentina). Participants were encouraged to expectorate and the procedure was discontinued after 3 ml of sputum has been obtained.

### Induced sputum processing

SI samples were transported at 4°C to the TB containment level-3 research laboratory at Universidad Peruana Cayetano Heredia (Lima, Peru). One aliquot was used for auramine smear microscopy and mycobacterial culture/direct drug susceptibility testing by the MODS method [19]. The remaining sample was centrifuged at 430 g to remove cellular debris, and then the supernatant was filtered through a 0.2 µM Durapore membrane (Millipore, Billerica, MA, USA). This protocol does not remove MMPs [20]. Samples were aliquoted and frozen at −20C analysed for MMP activity.

Concentrations of MMP-1, -2, -3, -7, -8, -9 were analysed by Luminex multiplex array (R&D Systems, Minneapolis, MN, USA). TIMP-1 and TIMP-2 were analyzed by ELISA kit (R&D Systems, Minneapolis, MN, USA). Total protein concentration was measured by Bradford assay [21] (Sigma-Aldrich, St Louis, MO, USA).

### Statistical analysis

Statistical analyses were performed using PRISM Version 5 (GraphPad, La Jolla, CA, US) and STATA 12 (Stata Corp., College Station, TX, USA). Parametric data are presented as mean and standard deviation (SD) and nonparametric data as median and interquartile range (IQR). Analysis between groups was by unpaired t test for parametric data and by Mann-Whitney U test for non-parametric data. Ordered logistic regression was used to evaluate association between TB Score and MMP concentrations. Correlation between MMPs was assessed by Spearman analysis. The non-parametric Friedman test and Dunn's multiple comparison post hoc test was used to compare differences between visits [22]. A p<0.05 was considered significant.

### Ethics Statement

This study received Institutional Review Board approval from Universidad Peruana Cayetano Heredia, Lima, Peru and the Peruvian Ministry of Health (DISA Lima Este). Written informed consent was received from all participants and all data was processed anonymously.

## Results

### Cross Sectional Study

**Figure 1:**
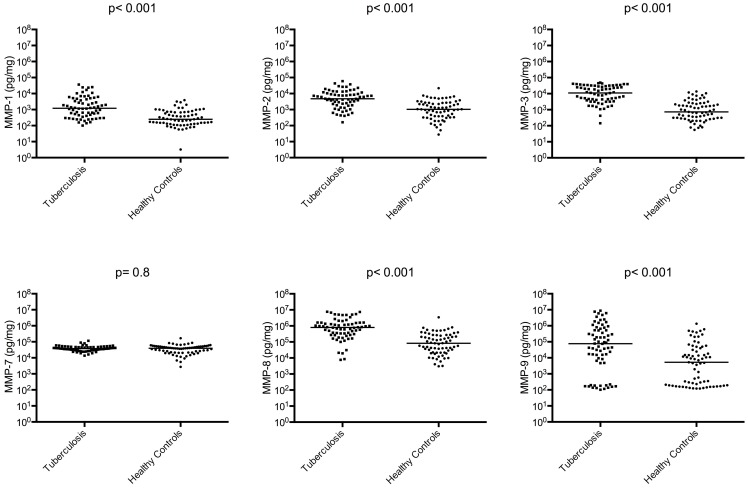
Multiple MMP concentrations are increased in induced sputum from TB patients. MMP-1, MMP-2, MMP-3, MMP-8, MMP-9 concentrations are significantly increased among TB patients compared to healthy controls analysed at time of TB diagnosis. The horizontal line is the median value.

**Figure 2:**
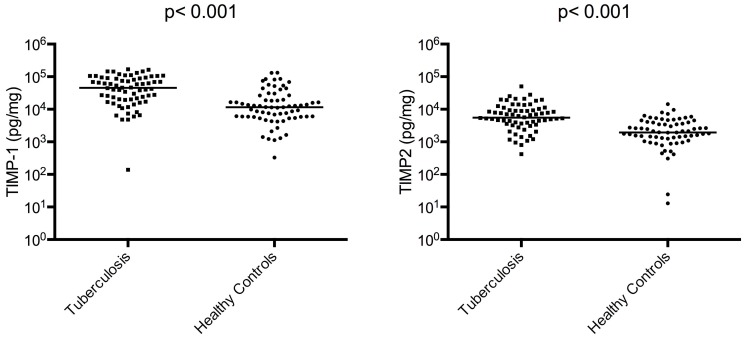
TIMP-1/2 concentrations are elevated in TB patients compared to controls. TIMP-1 and TIMP-2 concentrations are increased in induced sputum from TB patients compared to healthy controls analysed at time of TB diagnosis. The horizontal line is the median value.

We enrolled 68 TB participants and 69 healthy controls. The demographic and clinical characteristics are presented in Table 1. All patients had drug-sensitive TB. There were more males in the TB patient group than in the control group but gender was not significantly associated with MMP or TIMP concentrations overall or within groups when analysed by Mann-Whitney test. MMP-1, -2, -3, -8, and -9 concentrations were increased in TB patients compared to controls as were TIMP-1/2 concentrations (p<0.001 for each variable; Figure 1 and Figure 2). MMP-7 was no different between TB patients and control subjects. In TB patients, sputum MMP-3 and -9 concentrations were increased 15.2 fold and 14.4 fold respectively which were the greatest fold increases compared to controls. We investigated correlations between MMPs and TIMPS, and demonstrated correlations between the gelatinases MMP-2 and -9 (Rho = 0.83; p<0.001), between MMP-1 and its activator MMP-3 (Rho = 0.64; p<0.001). TIMP-1 concentrations correlated with MMP-8 (Rho = 0.71; p<0.001) and TIMP-2 concentrations with TIMP-1 (Rho = 0.74; p<0.001), MMP-2 (Rho = 0.66; p<0.001), -8 (Rho = 0.82; p<0.001) and -9 (Rho = 0.61; p<0.001).

**Table 1:** Characteristics of the study population.

Characteristics	Healthy Participants (n = 69)	Tuberculosis Participants (n = 68)	P-value (*)
Male (%)	12 (17.4%)	42 (61.8%)	<0.001
Mean Age (SD)	32.7 (12.6)	31.1 (13.8)	NS
Mean BMI (SD)	25.6 (3.8)	21.0 (2.9)	<0.001
Mean Temperature (SD)	36.2 (0.2)	36.6 (0.6)	<0.001
Mean TBscore (SD)	0.2 (0.4)	5.6 (1.9)	<0.001
Cough (%)	1 (1.5%)	63 (92.7%)	<0.001
Haemoptysis (%)	0 (0%)	30 (44.1%)	<0.001
Dyspnoea (%)	0 (0%)	37 (54.4%)	<0.001
Chest pain (%)	0 (0%)	36 (52.9%)	<0.001
Night sweating (%)	0 (0%)	42 (61.8%)	<0.001
Pale conjunctivae (%)	5 (7.3%)	61 (89.7%)	<0.001
Tachycardia (%)	1 (1.5%)	20 (29.4%)	<0.001
Abnormal lung examination (%)	2 (2.9%)	61 (89.7%)	<0.001
Axillary Temperature >37 C	0 (0%)	9 (13.2%)	0.002
Body mass index (BMI)			0.01
BMI<18	0 (0%)	5 (7.35%)	
BMI<16	0 (0%)	3 (4.4%)	
Middle upper arm circumference (MUAC)			0.05
MUAC <22 cm	3 (4.4%)	8 (11.8%)	
MUAC <20 cm	0 (0%)	3 (4.4%)	

* T test for differences of means; *X^2^* for difference in proportions.

TBscore is drive all clinical and anthropometric data presented in the table.

NS = not significant.

**Figure 3:**
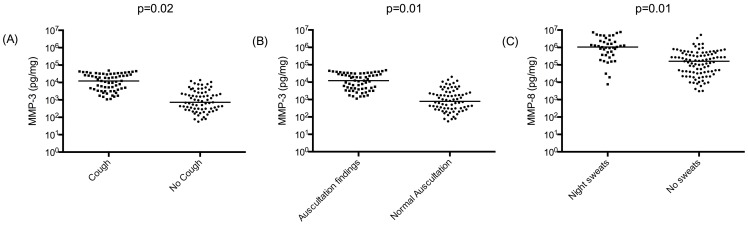
Clinical characteristics and their correlation with MMP-3 and -8 concentrations. MMP-3 concentrations are increased among TB patients who presented with (A) cough and (B) positive findings on auscultation. (C) MMP-8 concentrations are increased among TB patients who reported night sweats.

Next, we analysed the relationship between clinical characteristics and sputum MMP concentrations. MMP-3 concentrations were elevated in patients with cough or with positive findings on auscultation, which may result from pulmonary inflammation (p = 0.02 and 0.01 respectively; Figure 3A/B).. MMP-8 concentrations were increased in TB patients with night sweats (p = 0.01, Figure 3C). There was no statistically significant difference in MMPs and TIMPs concentrations in patients who had dyspnoea, chest pain or haemoptysis compared to those who did not. MMP and TIMP concentrations did not correlate with body mass index (BMI), temperature or age. MMP-3 had the best correlation with CXR score (Rho = 0.63; p<0.01).

To investigate the relationship of MMP/TIMP concentrations with the clinical disease severity score, we developed an ordered logistic regression model. The TB Score was categorized in 3 equal percentiles (≤5; >5 and <7; ≥7). Univariate analysis showed no association between MMP or TIMP concentrations with TB Score. In the multivariate analysis, after adjustment for age, gender, MMPs and TIMPs, MMP-1 and -3 were independently associated with TB Score (p<0.05).

### Longitudinal Cohort Study

**Figure 4:**
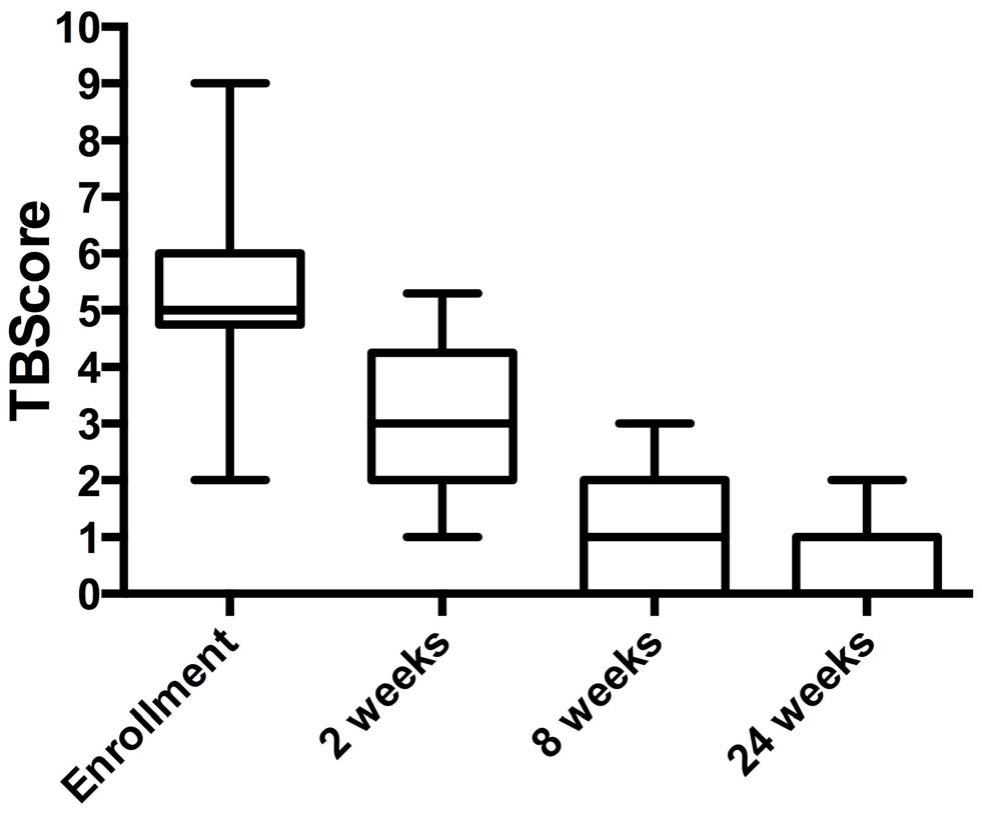
Median TBscore falls in patients over the course of the study. There is a significant decrease of TBscore comparing 2^nd^, 8^th^ and 24^th^ weeks with enrolment (p<0.05).

From the 68 patients with TB, 46 were followed-up longitudinally during the course of treatment until cure. There were no statistically significant differences in baseline characteristics between cross-sectional and cohort TB patients. Fifty-seven percent of patients were culture negative at the first follow-up visit at 2 weeks and 100% by the 8-week review, and all remained culture negative until the end of treatment. Median (IQR) TB Scores at enrolment, 2^nd^, 8^th^ and 24^th^ weeks were 5(6), 3(4), 1(2) and 0(1) respectively showing clinical improvement (Figure 4).

**Figure 5:**
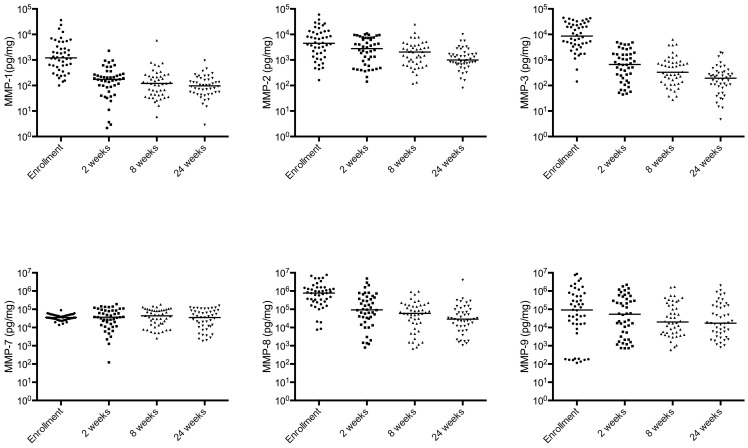
Sputum MMP concentrations decrease during TB treatment. 46 patients were followed longitudinally and induced sputum was collected at enrolment, weeks 2, 8 (at the end of the intensive treatment phase) and at the end of treatment (24 weeks). MMP-1, -2, -3, -8 and -9 concentrations significantly decreased during the course of treatment.

**Figure 6:**
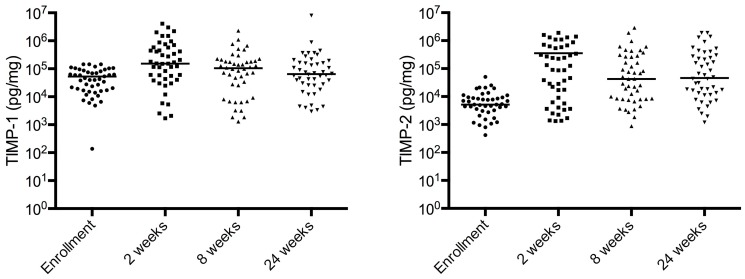
TIMP concentrations initially rise during TB treatment. In contrast to the decline in MMP concentrations, TIMP-1 and TIMP-2 concentrations significantly increase in the first two weeks of treatment, indicating a switch from a matrix degrading to matrix protective environment. Subsequently TIMP-1/2 concentrations decrease.

MMP concentrations decreased during TB treatment (Figure 5). MMP-1, MMP-3 and MMP-8 concentrations were significantly different at 2^nd^, 8^th^ and 24^th^ weeks compared with baseline (p<0.001). The decrease in MMP-2 and MMP-3 concentrations between 2 and 24 weeks was significant (p<0.001). In contrast, TIMP-1 and -2 concentrations were significantly increased at 2 and 8 weeks compared to concentrations at the start of TB therapy (p<0.05). TIMP-2 concentrations at 24^th^ week were significantly increased compared with baseline (p<0.001). TIMP concentrations decreased after 2^nd^ week, but did not fall below values at the enrolment visit (Figure 6).

### MMPs and TB culture conversion

**Figure 7:**
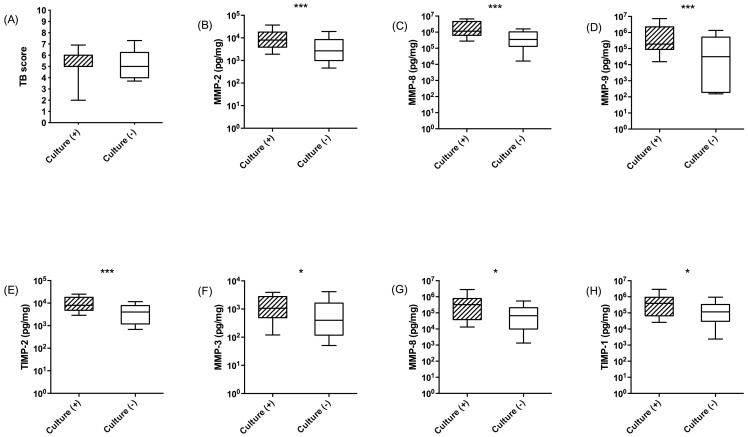
MMP and TIMP concentrations at admission and 2 weeks into therapy correlate with sputum culture conversion at 2 weeks. (A) TBScore is not significantly different at baseline between sputum converters and non-converters. MMP-2, MMP-8 and MMP-9 and TIMP-2 (B–E) concentrations at diagnosis are higher in patients who remain culture positive at 2 weeks (shaded boxes) than those who culture convert (open boxes). At 2 weeks of treatment, MMP-3, MMP-8 and TIMP-1 (F–H) concentrations are elevated in patients who remain culture positive compared to those who are culture negative. Boxes indicate 25 and 75 centile with median value; whiskers are 10–90 centiles. (*) p<0.05 (**) p<0.01 and (***) p<0.001.

Next, we analysed patients according to whether they became sputum culture negative at 2 weeks or not (culture conversion). Culture conversion was not related to TBscore at baseline (Figure 7A). MMP-2, MMP-8, MMP-9 and TIMP-2 concentrations at beginning of TB treatment were significantly higher among patients who remained culture positive at 2^nd^ week (p<0.01 Figure 7B–E). At week 2, MMP-3, MMP-8 and TIMP-1 concentrations remained higher amongst those who were culture positive compared with culture negative patients (p<0.05; Figure 7F–H).

## Discussion

In this first longitudinal analysis of a carefully characterised cohort of TB patients, we demonstrate that multiple MMP and TIMP concentrations are increased in TB patients at presentation, and that MMP-1 and -3 correlated most closely with the TB severity. As patients clinically improve, MMP-1, -3, and -8 concentrations decrease rapidly during treatment. In contrast, TIMP-1/2 concentrations increase, indicating some resolution of the matrix-degrading phenotype in which increased MMP activity is relatively unopposed by TIMPs [23].

In this patient population, MMP-1, -2, -3, -8 and -9 concentrations as well as TIMP-1/2 concentrations were markedly elevated in TB patients. These data are consistent with previous findings from ourselves and others [4], [17], [24] although the key role of MMP-8 which is secreted by diverse cells including neutrophils, increasingly recognised as a key cell in the immune response to TB [25], [26], has not been previously defined. Immunoreactive MMP concentrations were analyzed by Luminex array, and we have previously shown that this corresponds to MMP activity analyzed by casein zymography [4]. MMP-1 and -3 concentrations were correlated most consistently with clinical findings including overall TB score and may reflect the extent of pulmonary tissue destruction. Although MMP-9 concentrations were significantly elevated in patients with TB, there was significant overlap and some patients had levels equivalent to controls, which may reflect biological variability in airway MMP concentrations.. MMPs with similar functions such as the gelatinases MMP-2 and -9 correlated closely, as did the stromelysin MMP-3 with the collagenase MMP-1, which may form part of a proteolytic cascade [27]. TIMP concentrations also correlate with various MMPs but whether this is because they are co-secreted is unknown.

The longitudinal study has revealed that as patients respond to treatment, MMP-1, -3 and -8 concentrations fall rapidly within 2 weeks although all MMP concentrations continued to further decrease over the study period. However, even at the end of TB treatment, MMP concentrations remain elevated, which is consistent with data on pro-inflammatory chemokines [28]. Together with the elevated TIMP-1/2 concentrations observed at 6 months, this may indicate that tissue remodelling continues which may be one reason that some patients develop fibrotic lung disease following infection.

An interesting observation was the increase in TIMP-1/2 concentrations 2 weeks into TB treatment. We have previously proposed that active TB is characterised by a matrix degrading phenotype [8], [29] in which MMP activity is relatively unopposed by inhibitors. It may be that early in the resolution phase of acute TB a rise in TIMP secretion matched by a fall in MMP release is the host response to reverse the matrix degrading phenotype. This increase in TIMPs with a concurrent suppression of MMPs may help preserve lung matrix and facilitate the remodeling of the lung after the extensive destruction driven by pulmonary TB.

The TBscore at the start of treatment did not differ between patients who subsequently become TB sputum culture negative or not. However, those patients who remain culture positive at 2^nd^ week had higher sputum concentrations of MMP-2, -8 and -9 at baseline and of MMP-3, -8 and -9 at 2^nd^ week. TIMP-2 was similarly elevated. This may suggest that increased sputum TB load is associated with increased tissue destruction. Possibly these mediators may be useful components of a biomarker panel for active TB [30] or MMPs may be predictors of poor response to treatment. In order to more fully explore this, further studies of MDR-TB patients and others not responding to treatment is required.

In conclusion, MMPs particularly MMP-1 and its activator MMP-3 are elevated in patients with TB and relate to clinical symptoms. MMP-1, -3 and -8 concentrations fall sharply with treatment and at the same time there is an associated increase in inhibitor TIMP concentrations, which may reflect resolution of the matrix degrading phenotype. At the end of successful therapy, MMP and TIMP activity remains elevated, which may be key in tissue remodelling and scarring that lead to long-term fibrosis. The data suggest an unrecognized role for MMP-8 that is persistently elevated and high concentrations are associated with more persistent sputum positivity. It is not clear whether this reflects neutrophil activity in TB and further data are also required to determine its role in tissue destruction. Our results suggest that specific MMPs may be a useful component of a biomarker panel for active TB with other clinical and immunological parameters. Further longitudinal studies as well as work in cellular and animal models exploring MMP activity in non-healing and drug-resistant disease will elucidate whether MMPs or their upstream regulators are potential therapeutic targets.
